# Benchmarking of the 2010 BioCreative Challenge III text-mining competition by the BioGRID and MINT interaction databases

**DOI:** 10.1186/1471-2105-12-S8-S8

**Published:** 2011-10-03

**Authors:** Andrew Chatr-aryamontri, Andrew Winter, Livia Perfetto, Leonardo Briganti, Luana Licata, Marta Iannuccelli, Luisa Castagnoli, Gianni Cesareni, Mike Tyers

**Affiliations:** 1 School of Biological Sciences, University of Edinburgh, Edinburgh EH9 3JR, UK; 2 Department of Biology, University of Rome Tor Vergata, Rome 00133, Italy; 3 IRCSS, Fondazione Santa Lucia, Rome 00143, Italy; 4 Center for Systems Biology, Samuel Lunenfeld Research Institute, Mount Sinai Hospital, Toronto, Canada

## Abstract

**Background:**

The vast amount of data published in the primary biomedical literature represents a challenge for the automated extraction and codification of individual data elements. Biological databases that rely solely on manual extraction by expert curators are unable to comprehensively annotate the information dispersed across the entire biomedical literature. The development of efficient tools based on natural language processing (NLP) systems is essential for the selection of relevant publications, identification of data attributes and partially automated annotation. One of the tasks of the Biocreative 2010 Challenge III was devoted to the evaluation of NLP systems developed to identify articles for curation and extraction of protein-protein interaction (PPI) data.

**Results:**

The Biocreative 2010 competition addressed three tasks: gene normalization, article classification and interaction method identification. The BioGRID and MINT protein interaction databases both participated in the generation of the test publication set for gene normalization, annotated the development and test sets for article classification, and curated the test set for interaction method classification. These test datasets served as a gold standard for the evaluation of data extraction algorithms.

**Conclusion:**

The development of efficient tools for extraction of PPI data is a necessary step to achieve full curation of the biomedical literature. NLP systems can in the first instance facilitate expert curation by refining the list of candidate publications that contain PPI data; more ambitiously, NLP approaches may be able to directly extract relevant information from full-text articles for rapid inspection by expert curators. Close collaboration between biological databases and NLP systems developers will continue to facilitate the long-term objectives of both disciplines.

## Background

Before the explosion of online data archives such as Medline and PubMed, searches of the scientific literature for specific data content was a tedious practice that relied on dedicated paper-based services such as Current Contents. With the advent of electronic text databases and Internet access, the entire corpus of biomedical literature can be readily queried by author name and free-text keywords, such as gene or disease names. Nevertheless, whilst retrieving the literature of interest is now a relatively trivial task, mining and archiving the individual biological data elements contained within each of the millions of publications is still not possible. De facto there is no well-validated procedure that enables extraction of relevant information from the biomedical literature by automated parsing algorithms. This situation exists for several reasons, not least because information is embedded in non-standard descriptive natural language. The problem is exacerbated by the fact that publication authors typically fail to use unambiguous identifiers to describe bio-molecular entities and by the fact that data, with few exceptions [1,2], are never summarized in a format that is easily readable by computers [3,4]. As a consequence most biomedical data is embedded in essentially unextractable form in the scientific literature.

**Figure 1:**
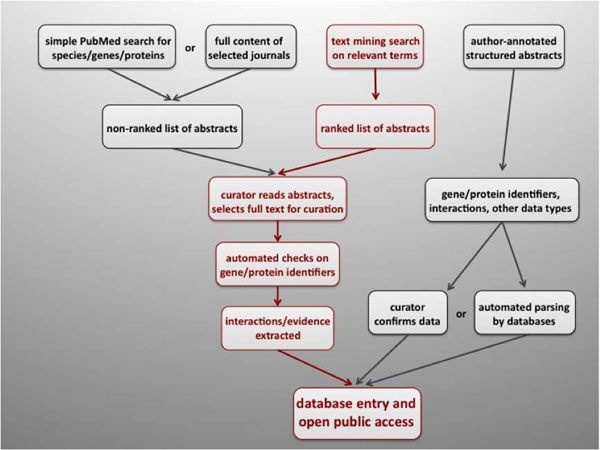
Summary of biocuration strategies. Text mining assisted work flow is shown in red.

To date, only the perseverance of expert curators at specialized biological databases enables a fraction of the available data to be accessed for automatic codification and computation. Manual curation, although more accurate and significantly more reliable than automated annotation [5], is a tremendously time-consuming practice that severely limits the number of articles that can be scrutinized and annotated. Although automated methods have been established to confirm gene/protein identities and assign structured evidence codes [6] the entire curation process relies on the judgment and input of expert curators at each step. An emerging alternative to full manual curation is the use of text mining tools, which can improve curation progress by the identification of relevant articles that contain data types of interest (Figure 1) [7-9].

High-throughput technologies have recently permitted the rapid accumulation of vast collections of genome-scale data for mRNA expression [10], protein post-translational modifications [11], protein-nucleic acid interactions, protein-protein interactions and genetic interactions [12-14]. These various molecular interactions are organized into complex networks that underlie all aspects of cellular structure and function. The possibility of deconstructing biological responses into constituent molecular interactions has motivated databases such as BioGRID [15,16] and MINT (Molecular INTeraction) [17,18] to undertake extraction and in-depth annotation of physical and genetic interactions reported in the primary literature. Once extracted and housed in an organized form, these interaction data enable computational analysis of biological networks, prediction of gene/protein function and the facile look-up of molecular interactions by biologists.

To date, however, these interaction database initiatives have relied exclusively on manual parsing and curation of the literature. Although complete coverage of the literature has been achieved for some model systems, notably budding and fission yeast [15,19], the vast majority of the literature remains untapped, particularly for human protein interactions. The scale of this problem is illustrated by the >11,000,000 publications on *H. sapiens* recorded in PubMed. Despite the cooperative efforts of protein interaction databases through the International Molecular Exchange (IMEx) consortium [15,18,20-27], whose purpose is to optimize the available resources by avoiding curation redundancy among its affiliated partner databases, comprehensive annotation of protein and genetic interactions dispersed throughout the biomedical literature is far from complete. Indeed, the rate of publication in the primary literature currently exceeds the curation capacity of all databases combined.

The Critical Assessment of Information Extraction in Biology (BioCreative) [28-31] initiative aims to evaluate state-of-the-art information extraction systems in biomedicine. In order to contribute to and ultimately benefit from this initiative, the MINT and BioGRID databases have provided expert curation of benchmark test sets for the 2010 edition of the competition, called BioCreative III. A major objective of Biocreative III was to close the gap between applications and end-users by encouraging the development of tools that meet the practical needs of database curators in the extraction of relevant data.

**Figure 2:**
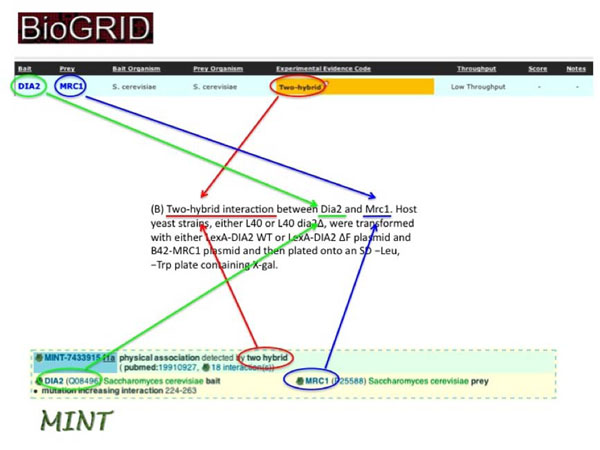
An example of annotation in BioGRID and MINT.

BioGRID and MINT annotate only data that is explicitly corroborated by experimental evidence reported in the peer-reviewed literature. MINT primarily annotates protein-protein interactions (PPI), whereas BioGRID annotates both protein and genetic interactions. While both databases are members of the IMEx consortium, MINT as active member and BioGRID as an observer, the two databases adhere to slightly different curation standards. MINT annotates interaction data according to the PSI-MI (Proteomics Standards Initiative–Molecular Interactions) controlled vocabulary developed and maintained by a working group of the Human Proteome Organization Proteomics Standards Initiative (HUPO-PSI) [32]. BioGRID employs an independently developed set of structured evidence codes for genetic and protein interactions [19], which are nevertheless largely re-mappable to the PSI-MI ontology [33]. BioGRID annotates the minimal information required for reporting a molecular interaction in accordance with the MIMIx (Minimum Information for a Molecular Interaction experiment) guidelines [34], whereas MINT endeavors to capture as many experimental details as possible within the PSI-MI structure [32] (Figure 2). Here, we describe the BioGRID and MINT contributions to the BioCreative III 2010 challenge. Both databases collaborated to provide manually curated datasets and expert knowledge that served as reference set for the evaluation of the various systems submitted by BioCreative participants for biomedical literature classification and extraction tasks.

## Results

BioGRID and MINT contributed high quality manually curated datasets as gold standards for three tasks of the Biocreative III Challenge, namely gene normalization (GN), article classification (ACT) and interaction method (IMT). The BioGRID dataset was curated from a pre-selected collection of publications provided by the Biocreative organizers while the MINT dataset was derived in the course of routine curation for the database. The aforementioned datasets formed the competition test set. The assembled data was revealed at the close of the competition.

### Gene normalization task

Gene normalization is the process of linking genes or proteins to stable database identifiers and as such is a crucial step in the annotation of biological interactions. Expert curators from both BioGRID and MINT participated with curators from other databases in the annotation of the test set for the gene normalization task. Curation specifications were set by the BioCreative III organizers and, for each gene mentioned in the full-text, required the annotation of taxon and Entrez Gene identifier. If either of these conditions could not be met, the gene was not annotated.

### Article classification task

From previous Biocreative editions, it has clearly emerged that classification of publication relevance for PPI data requires the analysis of full-text articles rather than abstracts [35]. Indeed, often an abstract will not contain the correct combination of key words or sentence that would otherwise allow classification of an article as containing interaction data. Thus any text-mining analysis based only on abstracts engenders frequent misclassification with a high rate of false positives. In fact, curators must often inspect the full-text of a publication to determine its relevance for interaction data. Even more problematically, in many instances there is no explicit statement contained anywhere in an article that describes an interaction, even if the interaction is actually demonstrated in the article. In these instances, curators must themselves infer and record the evidence for an interaction. For instance, positive experimental controls for interactions are rarely mentioned in the text, and results from medium or high-throughput experiments are usually reported in additional data files.

Unfortunately, full-text articles are often not accessible to text mining tools, in contrast to abstracts, which are freely available through PubMed in a common XML format. While open access initiatives have gained momentum, particularly as supported by the NIH, HHMI and Wellcome Trust, full-text articles are typically not freely available from for-profit high impact journals. In addition, XML specifications differ from one journal to another, requiring the development of specific tools to parse articles gathered from various publishers.

The BioCreative consortium organized an article classification task in order to assess the capability of available systems to classify pertinence of articles for PPI data based solely on abstracts. Participants were provided with a collection of recent abstracts, where for many of them free full-text articles were available. Systems were then tested for their ability to carry out a binary classification for relevance to PPI data, and were evaluated by comparing to manual curation results.

BioGRID (one curator) and MINT (two curators) manually classified a development and a test set of for relevance to PPI. Although the two databases do not share the same evidence codes and annotation vocabularies, both adopt the same rules for articles selection. Articles were considered suitable for curation only if the abstract suggested the presence of at least one experimentally verified protein interaction. As a consequence indirect functional connections or predicted interactions were not considered for positive classification. The resulting datasets were used as a benchmark to evaluate the precision of the dataset generated by the organizers for the training and test phases of the competition.

As the datasets annotated by BioGRID and MINT were partly overlapping (200 articles in common), it was possible to assess the inter-annotation agreement between the two databases. The percentage overlap between independent MINT and BioGRID curation was 95%, a remarkably high value given the different strategies and diverse expertise of different curators. Further analysis showed that the residual 5% discrepancy was not due to classification error per se, but rather due to contextual ambiguities in the abstract. For instance, in one of the scrutinized articles (PMID:19628465) a phosphorylation event was cited in the abstract even though no experimental evidence was proved.

### Interaction methods task

**Figure 3:**
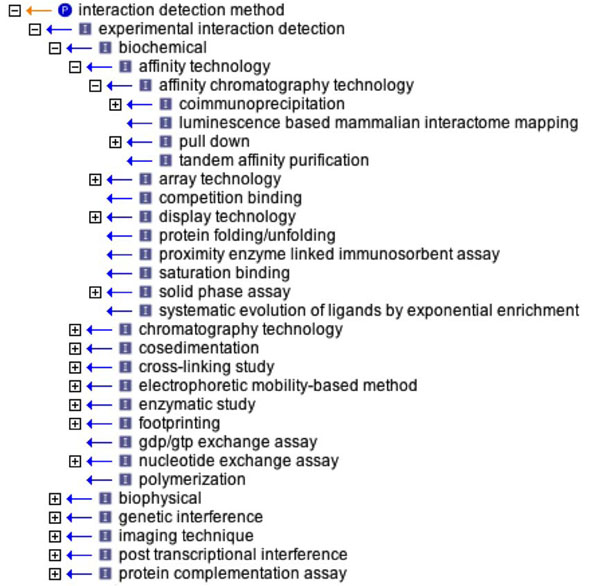
An overview of the Interaction Detection Methods branch in PSI-MI ontology.

A crucial aspect in the annotation of PPI data is the determination of the experimental method used to support the interaction. The reliability of any given interaction is correlated with the accumulation of experimental evidence obtained by diverse techniques [36]. The PSI-MI standard is based on a rich but well-controlled vocabulary that permits a deep and granular description of the experimental methods employed in protein interaction analysis (Figure 3). The PSI-MI ontology served as the basis for the interaction method task where participants were assigned with providing a ranked list of interaction pairs associated to the method used for their identification. For each interaction, multiple methods could be assigned, as supported by the article text.

Both BioGRID and MINT annotated physical interaction and co-localization evidence, BioGRID in accordance with the MIMIx recommendations and MINT in accordance with the IMEx curation guidelines [37]. The diversity in annotation details did not affect the identification of interaction pairs or the annotation of the experimental method, but only the extent of experimental detail recorded, such as particular interaction domains within a protein sequence or mutations that affected the interaction. For the description of the experimental method, curators from both databases selected the deepest term available in the PSI-MI ontology.

The BioGRID test set was composed of protein-protein interactions extracted from articles published in the journals *Embo Journal*, *EMBO Reports*, *Developmental Cell*, *Molecular Biology of the Cell*, *Molecular Cell*, *Molecular and Cellular Biology*, *Proceedings of the National Academy of Sciences*, *The Journal of Biological Chemistry* and *The Journal of Molecular Biology*. The MINT test set was composed of protein-protein interactions extracted from articles published in *EMBO Journal* and *EMBO Reports.* These manual curation efforts resulted in a dataset of 157articles that contained evidence for 954 interactions for BioGRID and 66 articles that contained evidence for 3093 interactions for MINT. The reason for the high number of interactions in the MINT dataset is due to the chance presence of articles that reported almost 2300 interactions derived from medium and high-throughput experiments (PMID:20467437, PMID:20508643, PMID:20467438, PMID:20508642).

Detailed results of comparisons between these manually annotated interaction datasets and interactions parsed automatically from the same publications are described elsewhere in this issue. Nevertheless, even from casual inspection, it is still possible to observe a substantial discrepancy between manual and automated curation [5,38], suggesting that current algorithms still need significant development and improvements.

## Conclusions

The advent of genomics has enabled the systematic description of entire genomes [39]. The next major challenge is the complete functional annotation of genomes, as witnessed by the number of efforts aiming at deciphering the function of coding and non-coding regions of the genome [40]. A strategy that has widely proven its efficacy in predicting uncharacterized gene/protein functions is the analysis of gene and protein interaction networks [41]. The role of any given gene is thus strongly predicted by its cohort of interaction partners [42,43]. These interactions have been identified traditionally in focused studies reported in the literature, and more recently by high throughput genetic and protein interaction surveys [14,44].

However, the comprehensive annotation of interaction maps is far from complete [45], both because the vast interaction space is largely still unexplored, particularly for human genes/proteins, and also because specialized interaction databases have to date been unable to completely harvest all data from the biomedical literature. Curation is a time-consuming and intensive process and, despite the federation of efforts across the IMEx consortium, the major interaction databases would need an unrealistic number of curators to fully annotate the past and present biomedical literature.

In the future, this problem may be largely solved by the adoption of rigorously structured scientific abstracts that contain author-annotated data attributes, including standard gene identifiers and interaction evidence codes. Interaction data may then be captured automatically by various databases. Nascent efforts are underway to develop and implement computable abstracts as a new aspect of the scientific literature [4]. In the absence of a coordinated initiative by authors and journals to facilitate the annotation process, reliable text mining approaches will necessarily form a key pillar of the curation enterprise.

At this juncture, the performance of current automated information extraction systems is not comparable with manual curation. Text-mining tools are thus still unable to reliably capture the richness of experimental details from full-text articles and associated figures, tables and supplementary data nearly as effectively as human curators. Nevertheless text mining is placed to play an increasingly important role in improving the efficiency of manual curation by assisting the selection of relevant articles and facilitating the information extraction process.

On these premises, the 2010 version of the BioCreative Challenge was shaped with the explicit aim of directing the development of text mining systems towards the immediate needs of biocurators. The correct assignment of gene/protein identifiers is a sine qua non of systematic curation and, although gene mention detection methods have high accuracy, automated approaches are still far from effectively achieving correct database identifier assignment. Although it is now clear that the most reliable results are attained by mining the full-text of articles, abstracts are frequently the only freely-available resource. Thus, the aim of the Article Classification Task was the development of tools that would permit curators to obtain a more refined list of articles than from, for example, a simple PubMed query. Moreover, through parsing abstracts, available text mining systems are able to place each retrieved article in rank order of likely relevance, thereby greatly assisting the curator in the selection of articles more likely to contain protein interaction data, or other data types.

As the annotation of the experimental method employed to detect the interaction is a crucial aspect of the curation pipeline, the purpose of the Interaction Method Task was to develop tools able to assist curators in assignment of experimental details. Although performance of current systems in this task was far from optimal, these initial efforts represent an important starting point for the delivery of more efficient tools that facilitate this key aspect of biocuration [46]. Refinement of automated approaches will expedite the inspection of articles by curators and help ensure that fundamental evidence codes are not overlooked.

The realization of high-performance user-oriented text mining systems will require ever-closer collaborations between tools developers and biological interaction databases. In particular, the assembly and release of high quality benchmark datasets will be crucial for the refinement of text-mining algorithms. It will be of particular interest to develop rule sets that enable the capture of more subtle textual features that define biological interactions and evidence codes. These rules in turn will help establish the basis for structured scientific abstracts that are implicitly machine-readable. The BioCreative Challenge III competition of 2010 demonstrates that alliances between text mining groups and protein interaction databases, such as BioGRID and MINT, facilitate the research interests of all, to the overal benefit of the biomedical research community. The advancement of information extraction tools should enable the goal of full literature curation of biological interactions to be achieved in a reasonable time frame.

## Materials and methods

### Article classification task

For the article classification task curators from BioGRID and MINT, assisted by MyMINER software [47], classified a development set of 725 abstracts (365 BioGRID and 360 MINT) and a test set of 573 abstracts (284 BioGRID and 289 MINT) provided by the BioCreative organizers. MyMINER is a web application that permits rapid binary classification of text format objects into pertinent and non pertinent categories.

### Interaction method task – test set

BioGRID and MINT annotated the test set to assist the Interaction Method Task. Both databases curated protein-protein interactions in accordance with the PSI-MI controlled vocabulary, choosing the deepest possible child term of PSI-MI controlled vocabulary root term ‘interaction detection method’. UniProtKB [48] identifiers were used as protein descriptors. Information about the experimental technique used to determine an interaction can be available in any section of an article (materials and methods, results, figure legends, tables, supplemental materials). Each publication may report one or more experimental methods, each of which may support one or more interactions. BioGRID curated articles were from *Embo Journal* (issue 22 from 2008), *EMBO Reports* (issue 5 from 2009), *Developmental Cell* (issues 2,3 from 2008), *Molecular Biology of the Cell* (issues 6, 7, 10, 12 from 2008; issues 1, 3, 4, 5, 9, 15, 16, 19, 20, 21, 22, 24 from 2009; issues 1, 4, 5 from 2010), *Molecular Cell* (issue 6 from 2008; issues 2,4,5 from 2009), *Molecular and Cellular Biology* (issues 12,13, 15, 18, 20, 21 from 2008; issues 1, 2, 4, 5, 6, 7, 8, 11, 12, 13, 15, 17, 18, 21 from 2009), *Proceedings of the National Academy of Sciences* (issues 7,8 from 2010), *The Journal of Biological Chemistry* (issues 23, 24, 27, 28, 30, 31, 33, 36, 37, 38, 39, 40, 42, 43, 44, 45, 46, 47, 48, 49, 50, 51 from 2008; issues 1, 2, 4, 5, 6, 7, 8, 9, 10, 11, 12, 13, 14, 15, 16, 17, 18, 19, 20, 27, 28, 36, 41, 42 from 2009; issues 5, 6, 10, 18 from 2010) and *The Journal of Molecular Biology* (issues 2,4 from 2008; issue 2 from 2009). MINT articles were chosen from issues of *EMBO Journal* (issues 2,4,6,7,8,9,10,11,12,13 from 2010) and *EMBO Reports* (issues 5,6,7 from 2010). Both datasets are available for download at http://www.biocreative.org/resources/corpora/biocreative-iii-corpus/.

BioGRID dataset is also available at http://thebiogrid.org/downloads/archives/Other%20Datasets/Biogrid_Biocreative_2010_IMT.txt.zip.

### Gene normalization task – test set

BioGRID and MINT curators annotated genes from articles provided by the Biocreative organizers (PMID:18398472, PMID:19393081, PMID:20502630, PMID:20502631). For each identified gene, the taxon and EntrezGene identifier were reported. This annotated dataset served as test set for the Gene Normalization task.

## List of abbreviations

ACT: Article Classification Task; GN: Gene Normalization, HHMI: Howard Hughes Medical Institute; HUPO: Human Proteome Organization; IMEx: International Molecular Exchange Consortium; IMT: Interaction Method Task; MI: Molecular Interaction; MINT: Molecular INTeraction; NIH: National Institute of Health; NLP: Natural Language Processing; PPI: Protein Protein Interaction; PSI: Proteomics Standards Initiative.

## Authors' contributions

AC, GC and MT conceptualized the project. AC wrote the manuscript with input from GC and MT. AC, AW, LP, LL, MI, LC, GC participated in curation.

## Competing interests

The authors declare that they have no competing interests.
